# Attenuation of cold stress-induced exacerbation of cardiac and adipose tissue pathology and metabolic disorders in a rat model of metabolic syndrome by the glucocorticoid receptor antagonist RU486

**DOI:** 10.1038/nutd.2016.14

**Published:** 2016-04-25

**Authors:** K Nagasawa, N Matsuura, Y Takeshita, S Ito, Y Sano, Y Yamada, A Uchinaka, T Murohara, K Nagata

**Affiliations:** 1 Department of Pathophysiological Laboratory Sciences, Nagoya, Japan; 2 Department of Cardiology, Nagoya University Graduate School of Medicine, Nagoya, Japan

## Abstract

**Objectives:**

Chronic stress affects the central nervous system as well as endocrine, metabolic and immune systems. However, the effects of cold stress on cardiovascular and metabolic disorders in metabolic syndrome (MetS) have remained unclear. We recently characterized DahlS.Z-*Lepr*^fa^/*Lepr*^fa^ (DS/obese) rats, derived from a cross between Dahl salt-sensitive and Zucker rats, as a new animal model of MetS. We have now investigated the effects of chronic cold stress and glucocorticoid receptor (GR) blockade on cardiac and adipose tissue pathology as well as on metabolic parameters in this model.

**Methods:**

DS/obese rats were exposed to cold stress (immersion in ice-cold water to a depth of 1–2 cm for 2 h per day) with or without subcutaneous injection of the GR antagonist RU486 (2 mg kg^−1^day^−1^) for 4 weeks beginning at 9 weeks of age. Age-matched homozygous lean (DahlS.Z-*Lepr*^+^/*Lepr*^+^) littermates served as a control.

**Results:**

Chronic cold stress exacerbated hypertension as well as left ventricular (LV) hypertrophy, fibrosis and diastolic dysfunction in DS/obese rats in a manner sensitive to RU486 treatment. Cold stress with or without RU486 did not affect body weight or fat mass. In contrast, cold stress further increased cardiac oxidative stress as well as macrophage infiltration and proinflammatory gene expression in LV and visceral fat tissue, with all of these effects being attenuated by RU486. Cold stress also further increased GR and 11β-hydroxysteroid dehydrogenase type 1 mRNA and protein abundance in LV and visceral adipose tissue, and these effects were again inhibited by RU486. In addition, RU486 ameliorated the stress-induced aggravation of dyslipidemia, glucose intolerance and insulin resistance in DS/obese rats.

**Conclusions:**

Our results implicate GR signaling in cold stress-induced exacerbation of cardiac and adipose tissue pathology as well as of abnormal glucose and lipid metabolism in a rat model of MetS.

## Introduction

Chronic stress can influence the central nervous system as well as endocrine, metabolic and immune systems in humans.^1^ Increased stress has also been found to parallel the morbidity of obesity.^2^ Abdominal obesity and exposure to chronic cold stress each have multiple effects and can trigger the development of metabolic syndrome (MetS) in animal models or humans, and they are therefore serious risk factors for cardiovascular disease and diabetes. Although the neural pathways of stress responses in the brain differ depending on the type of stressor, activation of the hypothalamic–pituitary–adrenal axis is thought to be a final common pathway.^3^

Cortisol in humans and corticosterone in rodents are the principal circulating glucocorticoids and are secreted under the control of the hypothalamic–pituitary–adrenal axis. Increased secretion of glucocorticoids, as occurs in Cushing's syndrome, leads to central obesity, hypertension, hyperlipidemia and glucose intolerance,^4^ and it has been implicated in the pathophysiology of MetS.^5^ 11β-Hydroxysteroid dehydrogenase type 1 (11β-HSD1), which acts as a reductase converting inactive cortisone (11-dehydrocorticosterone) into active cortisol (corticosterone), is highly expressed in liver, adipose tissue and skeletal muscle,^6^ and increased expression of this enzyme leads to glucocorticoid excess.^7^ Glucocorticoid action is largely mediated by ligand-induced activation of the glucocorticoid receptor (GR), which belongs to the nuclear receptor superfamily of ligand-dependent transcription factors,^8^ with the local concentration of glucocorticoids dictating GR activation.^5^

We previously established a new animal model of MetS, the DahlS.Z-*Lepr*^fa^/*Lepr*^fa^ (DS/obese) rat, by crossing Dahl salt-sensitive (DS) rats with Zucker rats harboring a missense mutation in the leptin receptor gene (*Lepr*). When fed a normal diet, DS/obese rats develop a phenotype, including hypertension and cardiac abnormalities, similar to MetS in humans. These cardiac abnormalities include left ventricular (LV) diastolic dysfunction as well as LV hypertrophy and fibrosis,^9^ and these changes are associated with increased cardiac oxidative stress and inflammation.^10^ We recently showed that activation of glucocorticoid–GR signaling may contribute to the pathophysiology of MetS and its associated complications in DS/obese rats.^11^ The role of the glucocorticoid–GR system in the effects of chronic stress on cardiac and adipose tissue pathology associated with MetS has remained unclear, however. We have now investigated the effects of chronic cold stress and GR blockade on cardiac and adipose tissue pathology as well as on abnormal glucose and lipid metabolism in DS/obese rats.

## Materials and methods

### Animals and experimental protocols

Animal experiments were approved by the Animal Experiment Committee of Nagoya University Graduate School of Medicine (Daiko district, approval nos. 025-026 and 026-009). Eight-week-old male inbred DS/obese rats and their male DahlS.Z-*Lepr*^+^/*Lepr*^+^ (DS/lean) littermates were obtained from Japan SLC (Hamamatsu, Japan) and were handled in accordance with the guidelines of Nagoya University Graduate School of Medicine as well as with the Guide for the Care and Use of Laboratory Animals (NIH publication no. 85-23, revised 1996). The animals were fed normal laboratory chow, and both the diet and tap water were provided *ad libitum* throughout the experimental period. DS/obese rats were left untreated (MetS group) or were exposed to cold stress (immersion in ice-cold water to a depth of 1–2 cm for 2 h per day beginning at 0900 hours) and injected subcutaneously either with RU486 at a dose of 2 mg kg^−1^ day^−1^ (MetS+CS+RU486 group) or with vehicle (MetS+CS group). The cold stress protocol was determined on the basis of results of a previous study^12^ and our preliminary experiments. RU486 (Mifepristone; Tokyo Chemical Industry Co., Ltd, Tokyo, Japan) was injected 30 min before the onset of cold stress, and its dose was determined on the basis of previous results.^13, 14^ Age-matched homozygous lean (DS/lean) littermates of DS/obese rats served as control animals (CONT group). Body weight as well as food and water intake were measured weekly. An oral glucose tolerance test and insulin tolerance test were performed as previously described.^11^ At 13 weeks of age, all animals were killed and the heart and both visceral (retroperitoneal, epididymal and mesenteric) and subcutaneous (inguinal) fat tissue were excised for analysis.

### Blood pressure measurement, echocardiography and hemodynamics

Systolic blood pressure (SBP) was measured weekly in conscious animals by tail-cuff plethysmography (BP-98A; Softron, Tokyo, Japan). At 13 weeks of age, all rats were anesthetized by intraperitoneal injection of ketamine (50 mg kg^−1^) and xylazine (10 mg kg^−1^) and were subjected to transthoracic echocardiography as previously described.^15^ After echocardiography, a 2 F micromanometer-tipped catheter (SPR-320; Millar Instruments, Houston, TX, USA) that had been calibrated relative to atmospheric pressure was inserted through the right carotid artery into the left ventricle for measurement of hemodynamic parameters.^16^

### Histology and immunohistochemistry

LV and visceral (retroperitoneal) fat tissue was fixed with ice-cold 4% paraformaldehyde for 48 h, embedded in paraffin and processed for histology as described previously.^15^ In brief, transverse sections were stained either with hematoxylin–eosin for routine histological examination or with Azan-Mallory solution for evaluation of fibrosis.^15^ To evaluate macrophage infiltration into the myocardium and adipose tissue, we performed immunostaining for the monocyte–macrophage marker CD68 with the paraffin-embedded sections.^10^ All image analysis was performed with NIH Scion Image software (Scion, Frederick, MD, USA).^17^

### Measurement of biochemical parameters

Blood was collected from the right carotid artery of rats that had been deprived of food overnight and anesthetized by intraperitoneal injection of sodium pentobarbital (50 mg kg^−1^). The serum concentration of glucose was measured with a routine enzymatic assay, and the concentrations of insulin and corticosterone in plasma were measured with the use of enzyme-linked immunosorbent assay kits from Morinaga Bioscience Institute (Yokohama, Japan) and Assaypro (St Charles, MO, USA), respectively. Serum levels of total cholesterol, low-density lipoprotein (LDL)-cholesterol, high-density lipoprotein (HDL)-cholesterol, triglyceride and free fatty acids were measured with routine enzymatic assays.

### Assay of superoxide production

Reduced nicotinamide adeninedinucleotidephosphate (NADPH)-dependent superoxide production by homogenates of freshly frozen LV tissue was measured with an assay based on lucigenin-enhanced chemiluminescence as described previously.^15^ Superoxide production in LV tissue sections was also evaluated by staining with dihydroethidium, and the average of dihydroethidium fluorescence intensity values was calculated with the use of NIH Image software (ImageJ).^18^

### Quantitative reverse transcription-PCR analysis

Total RNA was extracted from LV and visceral (retroperitoneal) fat tissue and was subjected to reverse transcription and real-time PCR analysis as described^19^ with specific primers for cDNAs encoding atrial natriuretic peptide,^20^ brain natriuretic peptide,^20^ collagen type I or type III,^21^ transforming growth factor-β1,^20^ monocyte chemoattractant protein-1,^22^ osteopontin,^22^ the p22^phox^,^23^ gp91^phox^,^23^ or Rac1^10^ subunits of NADPH oxidase, GR,^19^ or 11β-HSD1.^19^ Reagents for detection of human glyceraldehyde-3-phosphate dehydrogenase mRNA (Applied Biosystems, Foster City, CA, USA) were used to quantify rat glyceraldehyde-3-phosphate dehydrogenase mRNA as an internal standard.

### Immunoblot analysis

Total protein was isolated from LV and visceral (retroperitoneal) fat tissue and quantitated as described previously.^17^ Equal amounts of protein were subjected to sodium dodecyl sulfate-polyacrylamide gel electrophoresis, and the separated proteins were transferred onto a polyvinylidene difluoride membrane, as described previously.^24^ The membrane was incubated overnight at 4 °C with mouse monoclonal antibodies to GR, rabbit polyclonal antibodies to 11β-HSD1 or rabbit monoclonal antibodies to glyceraldehyde-3-phosphate dehydrogenase, as described previously.^11^ Detection and quantification of immune complexes were performed as described.^17^

### Statistical analysis

Data are presented as means±s.e.m. Differences among groups of rats at 13 weeks of age were assessed by one-way factorial analysis of variance; if a significant difference was detected, intergroup comparisons were performed with Fisher's multiple-comparison test. The time course of body weight, food intake, SBP and heart rate were compared among groups by two-way repeated-measures analysis of variance. A *P*-value of <0.05 was considered statistically significant.

## Results

### Physiological analysis

**Figure 1:**
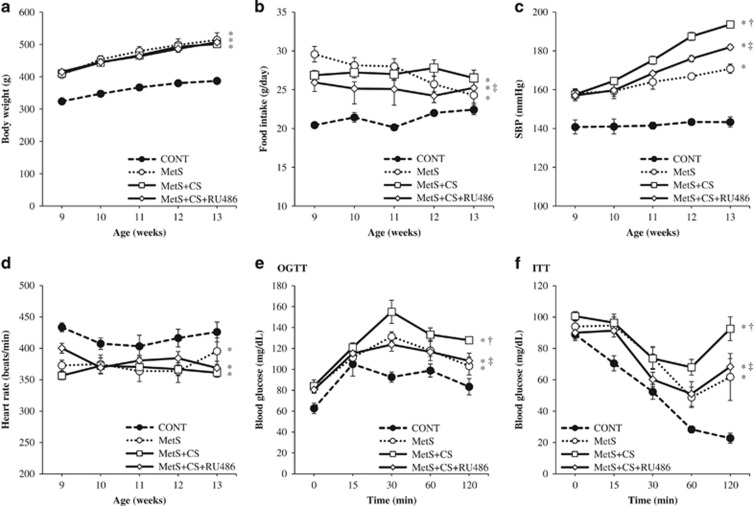
Time course of body weight (**a**), food intake (**b**), SBP (**c**) and heart rate (**d**) as well as the results of an oral glucose tolerance test (OGTT) (**e**) and insulin tolerance test (ITT) (**f**) performed at 13 weeks of age for rats in the four experimental groups. All data are means±s.e.m. (*n*=7, 7, 14 and 14 for CONT, MetS, MetS+CS and MetS+CS+RU486 groups, respectively). *P<0.05 vs CONT; ^†^*P*<0.05 vs MetS; ^‡^*P*<0.05 vs MetS+CS.

Body weight, food intake and SBP were significantly higher, whereas the heart rate was significantly lower, in the MetS group than in the CONT group (Figures 1a–d and Table 1). Cold stress and RU486 did not affect body weight or heart rate. Food intake was not changed in the MetS+CS group compared with the MetS group, but it was reduced in the MetS+CS+RU486 group compared with the MetS+CS group. In addition, SBP was further increased in the MetS+CS group compared with the MetS group, and this effect of cold stress was significantly attenuated by administration of RU486. At 13 weeks of age, the ratios of heart or LV weight to tibial length (indices of cardiac and LV hypertrophy, respectively) were significantly increased in the MetS group compared with the CONT group, and they were increased further in the MetS+CS group in a manner sensitive to RU486 treatment (Table 1). The ratios of visceral (retroperitoneal, epididymal or mesenteric) or subcutaneous (inguinal) fat weight to tibial length were increased in the MetS group compared with the CONT group, but were not affected further by cold stress or RU486 (Table 1). Echocardiography revealed that the interventricular septum thickness, LV posterior wall thickness, LV fractional shortening, LV ejection fraction, LV mass and relative wall thickness were all increased, whereas LV end-systolic dimension was decreased, in the MetS group compared with the CONT group. All of these increased parameters were further increased in the MetS+CS group compared with the MetS group in a manner sensitive to RU486 treatment (Table 1). The ratio of the peak flow velocity at the mitral level during rapid filling to that during atrial contraction (*E/A* ratio) was significantly reduced in the MetS group compared with the CONT group and was further reduced in the MetS+CS group, with this latter change being attenuated in the MetS+CS+RU486 group (Table 1). The deceleration time, isovolumic relaxation time and time constant of isovolumic relaxation as well as LV end-diastolic pressure and the ratio of LV end-diastolic pressure to the LV end-diastolic dimension were increased in the MetS group compared with the CONT group, and cold stress further increased these parameters in a manner sensitive to RU486 treatment (Table 1).

### Glucose tolerance, insulin sensitivity and metabolic parameters

The fasting serum glucose concentration was similar in the four experimental groups (Table 2). However, the fasting plasma insulin concentration was significantly increased in the MetS group compared with the CONT group, and it tended to be further increased in the MetS+CS group (*P*=0.052), with this latter change being significantly attenuated in the MetS+CS+RU486 group (Table 2). Moreover, an oral glucose tolerance test and insulin tolerance test revealed that cold stress exacerbated the glucose intolerance and insulin resistance apparent in the MetS group in a manner sensitive to RU486 (Figures 1e and f). The plasma concentration of corticosterone was similar in the MetS and CONT groups but it was increased in the MetS+CS and MetS+CS+RU486 groups compared with the MetS group (Table 2). Serum levels of total cholesterol, LDL-cholesterol, HDL-cholesterol, triglyceride and free fatty acids were significantly higher in the MetS group than in the CONT group. Total cholesterol was further increased in the MetS+CS group compared with the MetS group, whereas LDL-cholesterol, triglyceride and free fatty acids tended to be higher and HDL-cholesterol tended to be lower in the MetS+CS group than in the MetS group (*P* =0.75, 0.078, 0.069 and 0.075, respectively). All of these effects of cold stress were significantly attenuated by RU486 treatment (Table 2).

### Cardiomyocyte hypertrophy as well as cardiac fibrosis and gene expression

**Figure 2:**
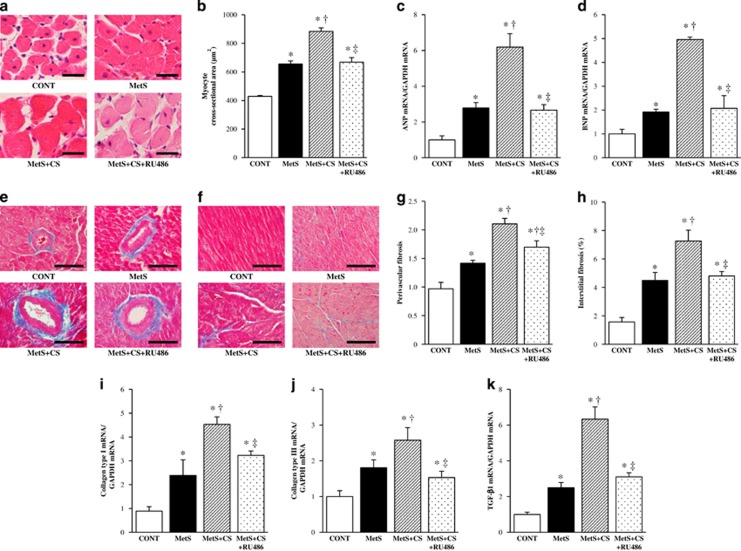
Cardiomyocyte size, expression of fetal-type cardiac genes, cardiac fibrosis and expression of fibrosis-related genes in the left ventricle of rats in the four experimental groups at 13 weeks of age. (**a**) Hematoxylin–eosin staining of transverse sections of the LV myocardium. Scale bars, 25 μm. (**b**) Cross-sectional area of cardiac myocytes determined from sections similar to those in (**a**). (**c**, **d**) Quantitative RT-PCR analysis of atrial natriuretic peptide (ANP) and brain natriuretic peptide (BNP) mRNAs, respectively. The amount of each mRNA was normalized by that of glyceraldehyde-3-phosphate dehydrogenase (GAPDH) mRNA and then expressed relative to the normalized value for the CONT group. (**e**, **f**) Collagen deposition as revealed by Azan-Mallory staining in perivascular and interstitial regions of the LV myocardium, respectively. Scale bars, 50 μm. (**g**, **h**) Relative extents of perivascular and interstitial fibrosis, respectively, as determined from sections similar to those in (**e**, **f**). (**i**–**k**) Quantitative RT-PCR analysis of collagen types I and III and transforming growth factor-β1 (TGF-β1) mRNAs, respectively. All quantitative data are means±s.e.m. (*n*=7, 7, 14 and 14 for CONT, MetS, MetS+CS and MetS+CS+RU486 groups, respectively). *P<0.05 vs CONT; ^†^*P*<0.05 vs MetS; ^‡^*P*<0.05 vs MetS+CS.

Microscopic analysis revealed that the cross-sectional area of cardiac myocytes was increased in the MetS group compared with the CONT group and was further increased in the MetS+CS group, with this latter change being attenuated in the MetS+CS+RU486 group (Figures 2a and b). Hemodynamic overload resulted in marked upregulation of the expression of fetal-type cardiac genes, including those for atrial natriuretic peptide and brain natriuretic peptide, in the MetS group. Cold stress further increased the expression of these genes in a manner sensitive to administration of RU486 (Figures 2c and d).

Azan-Mallory staining revealed that fibrosis in perivascular and interstitial regions of the LV myocardium was increased in the MetS group compared with the CONT group and was further increased in the MetS+CS group, with the effects of cold stress being attenuated in the MetS+CS+RU486 group (Figures 2e–h). The amounts of collagen types I and III and transforming growth factor-β1 mRNAs were also increased in the MetS group compared with the CONT group. Cold stress further increased the expression of these genes in a manner sensitive to RU486 treatment (Figures 2i–k).

### Cardiac inflammation

**Figure 3:**
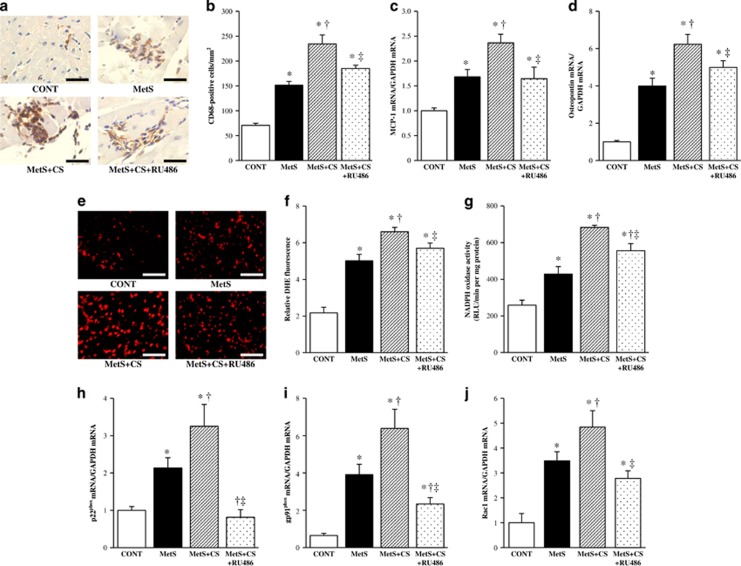
Macrophage infiltration, inflammatory gene expression, as well as NADPH oxidase activity and gene expression in the left ventricle of rats in the four experimental groups at 13 weeks of age. (**a**) Immunohistochemical analysis with antibodies to the monocyte–macrophage marker CD68. Scale bars, 50 μm. (**b**) Density of CD68-positive cells determined from sections similar to those in (**a**). (**c**, **d**) Quantitative RT-PCR analysis of monocyte chemoattractant protein-1 (MCP-1) and osteopontin mRNAs, respectively. The amount of each mRNA was normalized by that of glyceraldehyde-3-phosphate dehydrogenase (GAPDH) mRNA and then expressed relative to the normalized value for the CONT group. (**e**) Superoxide production as revealed by dihydroethidium staining in interstitial regions of the LV myocardium. Scale bars, 100 μm. (**f**) Relative dihydroethidium (DHE) fluorescence intensity determined from sections similar to those in (**e**). (**g**) NADPH-dependent superoxide production in LV tissue homogenates. Data are expressed as relative light units (RLU) per minute per milligram of protein. (**h**–**j**) Quantitative RT-PCR analysis of p22^phox^, gp91^phox^ and Rac1 mRNAs, respectively. All quantitative data are means±s.e.m. (*n*=7, 7, 14 and 14 for CONT, MetS, MetS+CS and MetS+CS+RU486 groups, respectively). *P<0.05 vs CONT; ^†^*P*<0.05 vs MetS; ^‡^*P*<0.05 vs MetS+CS.

Immunostaining for the monocyte–macrophage marker CD68 revealed that macrophage infiltration in the LV myocardium was increased in the MetS group compared with the CONT group and was further increased in the MetS+CS group, with this effect of cold stress being attenuated by the administration of RU486 (Figures 3a and b). The expression of monocyte chemoattractant protein-1 and osteopontin genes in the left ventricle of animals in the four groups showed a pattern similar to that of macrophage infiltration (Figures 3c and d).

### Cardiac oxidative stress

Superoxide production in myocardial tissue sections, as revealed by staining with dihydroethidium, as well as the activity of NADPH oxidase in homogenates of LV tissue were significantly increased in the MetS group compared with the CONT group. Cold stress further increased these parameters in a manner sensitive to RU486 treatment (Figures 3e–g). Cardiac expression of genes for the p22phox and gp91phox membrane components and for the Rac1 cytosolic component of NADPH oxidase in animals of the four groups showed a pattern similar to that for superoxide production (Figures 3h–j).

### Cardiac glucocorticoid-related gene expression

**Figure 4:**
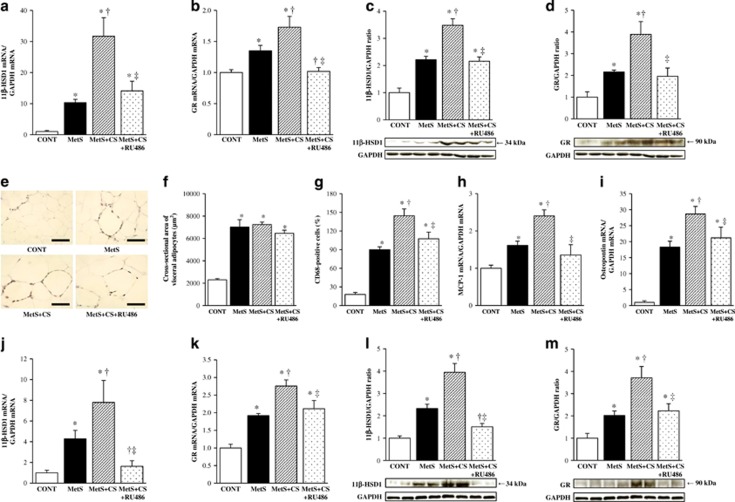
Glucocorticoid-related gene and protein expression in the left ventricle as well as macrophage infiltration and inflammatory gene and glucocorticoid-related gene and protein expression in visceral (retroperitoneal) adipose tissue of rats in the four experimental groups at 13 weeks of age. (**a**, **b**) Quantitative RT-PCR analysis of 11β-HSD1 and GR mRNAs in the left ventricle, respectively. The amount of each mRNA was normalized by that of glyceraldehyde-3-phosphate dehydrogenase (GAPDH) mRNA and then expressed relative to the normalized value for the CONT group. (**c**, **d**) Immunoblot analysis of 11β-HSD1 and GR proteins in the left ventricle, respectively. Representative immunoblots as well as the relative ratio of the amount of 11β-HSD1 or GR to that of GAPDH are shown. (**e**) Immunohistochemical analysis of visceral adipose tissue with antibodies to the monocyte–macrophage marker CD68. Scale bars, 100 μm. (**f**) Cross-sectional area of adipocytes determined from sections similar to those in (**e**). (**g**) The number of nuclei for CD68-positive cells as a percentage of total nuclei was determined from sections similar to those in (**e**). (**h**–**k**) Quantitative RT-PCR analysis of monocyte chemoattractant protein-1 (MCP-1), osteopontin, 11β-HSD1 and GR mRNAs in visceral adipose tissue, respectively. (**l**, **m**) Immunoblot analysis of 11β-HSD1 and GR proteins in visceral adipose tissue, respectively. All quantitative data are means±s.e.m. (*n*=7, 7, 14 and 14 for CONT, MetS, MetS+CS and MetS+CS+RU486 groups, respectively). *P<0.05 vs CONT; ^†^*P*<0.05 vs MetS; ^‡^*P*<0.05 vs MetS+CS.

Expression of 11β-HSD1 and GR at the mRNA (Figures 4a and b) and protein (Figures 4c and d) levels in the left ventricle was increased in the MetS group compared with the CONT group and was further increased in the MetS+CS group. These effects of cold stress were suppressed by RU486 treatment.

### Adipocyte hypertrophy as well as adipose tissue inflammation and gene expression

Hematoxylin–eosin staining and immunostaining for CD68 revealed that adipocyte cross-sectional area and macrophage infiltration in visceral adipose tissue were increased in the MetS group compared with the CONT group. Whereas adipocyte size was not affected by cold stress or RU486, macrophage infiltration was increased further by cold stress in a manner sensitive to administration of RU486 (Figures 4e–g). The expression of monocyte chemoattractant protein-1 and osteopontin genes in visceral adipose tissue was also increased in the MetS group compared with the CONT group and was further increased in the MetS+CS group, with these effects of cold stress being attenuated by RU486 (Figures 4h and i). Finally, expression of 11β-HSD1 and GR at the mRNA and protein levels in adipose tissue was increased in the MetS group compared with the CONT group, with cold stress further increasing such expression in a manner sensitive to RU486 treatment (Figures 4j–m).

## Discussion

We have here shown that chronic cold stress increased SBP, the plasma corticosterone concentration and glucocorticoid activity in the heart and adipose tissue as well as exacerbated LV hypertrophy, fibrosis, inflammation, oxidative stress, and diastolic dysfunction, adipose tissue inflammation, and abnormal glucose and lipid metabolism in DS/obese rats. Furthermore, all of these effects of cold stress were attenuated by treatment with the GR antagonist RU486. Our findings suggest that activation of glucocorticoid–GR signaling in the circulation as well as in the heart and adipose tissue may contribute to the stress-induced exacerbation of the pathophysiology of MetS and its associated complications.

Increased secretion of glucocorticoids due to pituitary adenoma in Cushing's syndrome causes central obesity, hypertension, hyperlipidemia and glucose intolerance.^4^ Such abnormalities are also associated with MetS. We have now shown that chronic cold stress increased the plasma corticosterone level as well as glucocorticoid activity in the heart and adipose tissue of DS/obese rats. Consistent with these findings, 11β-HSD1 expression in white adipose tissue was increased by stress in mice fed a high-fat, high-sugar diet, possibly contributing to local generation of active glucocorticoids within fat.^25^ RU486 did not affect the plasma corticosterone level of stressed DS/obese rats in the present study, but it attenuated the cold stress-induced enhancement of glucocorticoid–GR signaling in the heart and adipose tissue. The GR may therefore contribute to exacerbation of the pathophysiology of MetS and its associated complications by cold stress. Previous studies have also suggested a correlation between increased glucocorticoid activity and hypertension or metabolic disorders.^1, 26, 27, 28^

Hypertension due to glucocorticoid excess is well recognized.^14^ Insulin resistance and inflammation may also result in altered vascular function and thereby trigger hypertension.^29^ In addition, the production of reactive oxygen species by NADPH oxidase has been implicated in glucocorticoid-induced hypertension.^30, 31^ Consistent with these various observations, we found that the elevation of SBP induced by cold stress in DS/obese rats was associated with increased systemic glucocorticoid activity, insulin resistance, and cardiovascular inflammation and oxidative stress. RU486 attenuated the increase in SBP induced by cold stress in DS/obese rats, suggesting that GR signaling may play an important role in this effect of cold stress. In our previous study with unstressed DS/obese rats, in which the plasma corticosterone level was not increased in comparison with that in unstressed DS/lean rats, RU486 did not affect SBP in either rat strain.^11^ Although the reason for this apparent discrepancy is unclear, it is possible that the effect of RU486 on blood pressure depends on the level of glucocorticoid activity. RU486 has been shown to lower SBP in the mineralocorticoid-salt model of hypertension.^14^ The GR may therefore contribute in part to the development of hypertension in DS/obese rats, and RU486 may act more effectively in the setting of increased plasma corticosterone levels.

Increased activity of the sympathetic nervous system (SNS) also contributes to obesity-induced hypertension.^32^ However, obesity does not induce mass activation of the SNS. Instead, increased SNS activity is modest and appears to be differentially controlled in various tissues in obesity.^33^ Indeed, our previous data showed that urinary norepinephrine excretion did not increase and heart rate was reduced in unstressed DS/obese rats.^34^ These findings are consistent with previous results, showing that some rodent models of obesity have normal or reduced SNS activity and decreased blood pressure because of the disruption of central nervous system signaling pathways that link obesity with SNS.^32^ Leptin and other neuropeptides are possible links between obesity and hypertension. Meanwhile, chronic stress-induced activation of SNS can amplify obesity-induced pathology. However, since cold stress was intermittent (2 h per day) and measurements were made while the animals were not immersed in ice-cold water, sympathetic activity during measurements is unclear. Moreover, as the stressful stimulus was applied daily over the course of 4 weeks, the possibility arises that acclimatization to cold may have occurred to some degree, a well-recognized effect of cold on the SNS. It is thus difficult to precisely define the pathophysiological role of SNS in cold stress-induced exacerbation of cardiac and adipose tissue pathology and metabolic disorders in DS/obese rats. In contrast, our recent study showed that restraint stress also aggravated hypertension as well as increased urinary norepinephrine excretion in this model in a manner sensitive to propranolol.^34^ Different stressors induce differential activation of hypothalamic–pituitary–adrenal axis and SNS and both patterns and intensity of the associated cardiovascular changes may vary with the types of stress.

Obesity complicated with hypertension is associated with changes in cardiac structure and function.^35^ Cardiac hypertrophy represents an initial compensatory response of the heart to increased hemodynamic load, and direct activation of GR signaling in cardiomyocytes may promote pathological cardiac hypertrophy.^36, 37^ The effects of cold stress and RU486 on cardiac hypertrophy in the present study support this scenario. Increased circulating levels of proinflammatory cytokines can result in cardiac fibrosis and dysfunction.^38, 39^ Further increases in the levels of such cytokines induced by cold stress may have contributed to the exacerbation of cardiac injury in DS/obese rats. In addition, our data implicate GR signaling in the cold stress-induced exacerbation of cardiac fibrosis and diastolic dysfunction in DS/obese rats. We cannot rule out the possibility that blood pressure independently determined the effects of cold stress and RU486 on cardiac fibrosis and function. However, we previously found that RU486 ameliorated LV fibrosis and diastolic stiffness as well as attenuated LV oxidative stress and inflammation, without lowering blood pressure, in unstressed DS/obese rats.^11^

Cardiac inflammatory changes may contribute to myocardial fibrosis.^40^ We recently showed that macrophage infiltration into the interstitial space of the LV myocardium was accompanied by increased expression of genes for proinflammatory proteins such as monocyte chemoattractant protein-1 and osteopontin in the heart of DS/obese rats.^41^ The GR is expressed widely in the cardiovascular system, including the vessel wall, myocardium and inflammatory cells such as macrophages that invade vascular lesions.^42^ Glucocorticoids are also thought to exert immunoinhibitory actions. However, we have now found that GR blockade with RU486 attenuated cold stress-induced inflammatory responses in the heart and adipose tissue, suggesting that GR signaling may contribute to the stress-induced exacerbation of cardiac and adipose tissue inflammation. These findings are consistent with previous results, showing that RU486 attenuated inflammation in the cardiac interstitium as well as the upregulation of osteopontin mRNA abundance in vessel walls induced by deoxycorticosterone and salt.^14^ They are also consistent with the notion that glucocorticoids have both immunostimulatory and immunosuppressive functions,^43^ with GR signaling being mediated by different sets of transcription factors, coactivators and corepressors, allowing a shift in promoter activity depending on cellular activation status.^44^ We recently showed that the plasma corticosterone level is similar in DS/obese and DS/lean rats, and that RU486 attenuated cardiac and adipose tissue inflammation apparent in DS/obese rats,^11^ consistent with the notion that glucocorticoids are immunostimulatory within the normal physiological range of hypothalamic–pituitary–adrenal axis activity. The GR is recruited to the promoter of the Toll-like receptor 2 gene in cells stimulated with tumor necrosis factor-α and dexamethasone, with this interaction involving binding sites for the transcription factors STAT and NF-κB as well as the 3′ glucocorticoid response element.^45^ In contrast, glucocorticoids act in an immunoinhibitory manner when their levels are increased, as in chronically stressed animals.^46^ In the present study, the plasma concentration of corticosterone was slightly but not significantly (*P*=0.188) reduced in DS/obese rats compared with DS/lean rats, it was significantly increased by cold stress in DS/obese rats, but it did not differ between the CONT and MetS+CS groups. Together, our present and previous^11^ results suggest that glucocorticoids may exert proinflammatory effects via activation of the GR in this model of MetS both with and without chronic cold stress. Central or truncal obesity is linked to insulin resistance,^47^ and adipose tissue-specific amplification of glucocorticoid signaling induces all the characteristic features of MetS.^48, 49, 50^ We have now shown that the cold stress-induced augmentation of adipose tissue inflammation was associated with exacerbation of insulin resistance, consistent with previous findings.^51^ These observations suggest a possible role for GR signaling in macrophage recruitment in cardiac and adipose tissue as well as in insulin resistance. RU486 might therefore inhibit the augmentation of adipose tissue inflammation by cold stress in DS/obese rats by reducing glucocorticoid activity, leading to amelioration of glucose and lipid metabolic disorders.

In conclusion, chronic cold stress increased systemic and local glucocorticoid activity in the heart and adipose tissue as well as exacerbated hypertension, cardiac pathophysiology, adipose tissue inflammation and metabolic disorders in DS/obese rats. These effects of cold stress were attenuated by treatment with the GR antagonist RU486. Our findings thus suggest that activation of glucocorticoid–GR signaling may contribute to the cold stress-induced exacerbation of the pathophysiology of MetS and its associated complications. GR blockade may therefore prove effective for the treatment of MetS combined with chronic cold stress.

## Figures and Tables

**Table 1:** Physiological as well as cardiac, morphological and functional parameters of rats in the four experimental groups at 13 weeks of age

*Parameter*	*CONT*	*MetS*	*MetS+CS*	*MetS+CS+RU486*
Body weight (g)	387.1±4.4	513.9±29.1*	501.2±8.31*	506.8±7.9*
Food intake (g day^−1^)	22.4±0.6	24.3±3.3*	26.5.0±1.0*	25.2±1.0*^‡^
SBP (mmHg)	143.3±1.2	170.8±2.9*	193.6±2.6*^†^	181.9±2.5*^‡^
Heart rate (beats min^−1^)	426.0±9.8	395.5±8.9*	361.1±10.7*	368.7±11.5*
Heart weight/tibial length (mg mm^−1^)	30.2±0.5	36.4±1.2*	40.7±0.7*^†^	37.6±0.8*^‡^
LV weight/tibial length (mg mm^−1^)	22.0±0.5	26.9±1.2*	32.7±0.9*^†^	28.6±0.9*^‡^
Retroperitoneal fat weight/tibial length (mg mm^−1^)	100.0±5.6	469.1±30.1*	470.1±13.0*	468.8±9.6*
Epididymal fat weight/tibial length (mg mm^−1^)	126.4±6.0	413.6±49.6*	371.7±11.3*	390.5±13.1*
Mesenteric fat weight/tibial length (mg mm^−1^)	84.1±6.6	400.0±32.0*	388.7±17.0*	389.2±18.3*
Inguinal fat weight/tibial length (mg mm^−1^)	114.1±4.2	809.4±55.1*	817.4±33.9*	861.5±39.8*
IVST (mm)	1.57±0.03	2.02±0.13*	2.58±0.06*^†^	2.18±0.07*^‡^
LVPWT (mm)	1.43±0.06	1.91±0.11*	2.66±0.11*^†^	2.23±0.07*^†‡^
LVDd (mm)	7.79±0.21	7.40±0.32	6.82±0.09*^†^	7.22±0.11
LVDs (mm)	4.68±0.30	3.56±0.39*	3.15±0.11*	3.54±0.13*
LVFS (%)	35.8±2.1	52.4±4.5*	57.0±0.9*^†^	51.0±1.4*^‡^
LVEF (%)	70.4±0.7	83.8±3.3*	91.2±0.7*^†^	85.6±1.1*^‡^
LV mass (mg)	884.4±73.8	1190.1±101.4*	1578.1±91.3*^†^	1319.6±59.0*^‡^
RWT	0.43±0.03	0.58±0.06*	0.72±0.02*^†^	0.63±0.03*^‡^
*E/A*	1.69±0.06	1.52±0.06*	1.36±0.03*^†^	1.52±0.03*^‡^
DcT (ms)	41.6±1.9	55.1±3.7*	70.0±2.4*^†^	57.7±1.3*^‡^
IRT (ms)	24.1±0.8	32.5±2.9*	45.1±1.4*^†^	37.1±0.79*^†‡^
Tei index	0.38±0.01	0.49±0.04*	0.63±0.01*^†^	0.55±0.01*^†‡^
Tau (ms)	23.4±0.71	31.4±2.4*	43.9±6.2*^†^	32.4±4.5*^‡^
LVEDP (mmHg)	3.64±0.51	11.3±2.3*	23.2±3.1*^†^	15.2±1.8*^‡^
LVEDP/LVDd (mmHg mm^−1^)	0.40±0.04	1.45±0.31*	3.52±0.49*^†^	1.94±0.28*^‡^

Abbreviations: DcT, deceleration time; IRT, isovolumic relaxation time; IVST, interventricular septum thickness; LVDd, LV end-diastolic dimension; LVDs, LV end-systolic dimension; LVEDP, LV end-diastolic pressure; LVEF, LV ejection fraction; LVFS, LV fractional shortening; LVPWT, LV posterior wall thickness; RWT, relative wall thickness; tau, time constant of isovolumic relaxation. Data are means±s.e.m. (*n*=7, 7, 14 and 14 for CONT, MetS, MetS+CS and MetS+CS+RU486 groups, respectively). *P<0.05 vs CONT; ^†^*P*<0.05 vs MetS; ^‡^*P*<0.05 vs MetS+CS.

**Table 2:** Metabolic parameters of rats in the four experimental groups at 13 weeks of age

*Parameter*	*CONT*	*MetS*	*MetS+CS*	*MetS+CS+RU486*
Serum glucose (mg dl^−1^)	123.0±1.7	130.8±2.5	124.8±3.3	129.8±6.4
Plasma insulin (ng dl^−1^)	0.48±0.06	3.75±0.75*	5.87±1.67*	3.44±0.65*^‡^
Plasma corticosterone (ng ml^−1^)	812.5±62.5	528.7±46.6	1022.7±196.8^†^	1092.6±145.2^†^
Total cholesterol (mg dl^−1^)	75.2±3.1	291.8±32.4*	410.2±33.4*^†^	198.2±37.2*^†‡^
LDL-cholesterol (mg dl^−1^)	21.5±1.26	61.2±11.7*	91.2±14.3*	41.7±12.2^‡^
HDL-cholesterol (mg dl^−1^)	39.8±2.2	78.3±12.2*	57.0±8.4	82.5±4.1*^‡^
Triglyceride (mg dl^−1^)	61.2±6.7	1859.8±472.8*	2701.5±383.7*	767.0±201.1^†‡^
FFAs (mEq l^−1^)	0.83±0.04	1.37±0.22*	1.74±0.16*	1.25±0.06^‡^

Abbreviation: FFA, free fatty acid. Data are means±s.e.m. (*n*=7, 7, 14 and 14 for CONT, MetS, MetS+CS and MetS+CS+RU486 groups, respectively). *P<0.05 vs CONT; ^†^*P*<0.05 vs MetS; ^‡^*P*<0.05 vs MetS+CS.
